# Elbow Flexion Recovery and Respiratory Function in Total Traumatic Brachial Plexus Injury Patients Treated with Phrenic Nerve Transfer

**DOI:** 10.1055/s-0045-1811930

**Published:** 2025-11-21

**Authors:** Giovanni V.C. Guedes, Rogério R. Visconti, Rudolf N. Kobig, João A. M. Guimarães, Conrado T. Laett

**Affiliations:** 1 Neuromusuclar Research Laboratory, Teaching and Research Division, Instituto Nacional de Traumatologia e Ortopedia Jamil Haddad, Rio de Janeiro, RJ, Brazil

**Keywords:** brachial plexus/injuries, nerve transfer, neuronal plasticity, phrenic nerve, nervo frênico, plasticidade neuronal, plexo braquial/lesões, transferência de nervo

## Abstract

**Objective:**

To evaluate the outcomes of phrenic nerve transfer in total traumatic brachial plexus injury, focusing on elbow flexion and respiratory function.

**Methods:**

The present is a case series of 16 patients undergoing phrenic nerve transfer between 2014 and 2021. Patients over 18 years old, operated on for more than 6 months, and without other orthopedic conditions of the upper limb were included. Elbow flexion strength was assessed by Medical Research Council (MRC) scale and an isokinetic dynamometer, along with an electromyograph. Respiratory function was assessed by spirometry.

**Results:**

The patients were mainly young men affected by motorcycle accidents. Level III on the MRC was achieved by 37.5% of patients, with 43.8% reaching level IV. On average, elbow flexion strength was of 9.1% compared with that of the unaffected arm. The study identified inconsistent deficits in respiratory function, with no severe impairment in forced vital capacity and forced expiratory volume. Respiratory symptoms were not reported. Involuntary activation of the biceps brachii during forced respiratory cycles was observed, peaking after an initial recovery period.

**Conclusion:**

Phrenic nerve transfer effectively restored elbow flexion in most patients. We found signs of neuroplasticity that enhanced the motor control of the arm over time. We found no evidence of severe pulmonary impairment in these patients.

## Resumo

**Objetivo:**

Avaliar os resultados da transferência do nervo frênico em pacientes com lesão traumática total do plexo braquial, especialmente a flexão do cotovelo e a função respiratória.

**Métodos:**

Trata-se de uma série de casos de 16 pacientes submetidos à transferência do nervo frênico entre 2014 e 2021. Foram incluídos pacientes com mais de 18 anos, operados havia mais de 6 meses, e sem outras doenças ortopédicas do membro superior. A força de flexão do cotovelo foi avaliada pela escala do Medical Research Council (MRC), por dinamometria isocinética e por eletromiografia. A função respiratória foi analisada por espirometria.

**Resultados:**

Os pacientes eram principalmente homens jovens que sofreram acidentes com motocicletas. Na escala do MRC, 37,5% dos pacientes atingiram o nível III, e 43,8%, o nível IV. Em média, a força de flexão do cotovelo foi de 9,1% em comparação à do braço não acometido. O estudo identificou déficits inconsistentes na função respiratória, sem comprometimento grave da capacidade vital forçada e do volume expiratório forçado. Não houve relatos de sintomas respiratórios. A ativação involuntária do bíceps braquial foi observada durante ciclos respiratórios forçados, com pico após um período inicial de recuperação.

**Conclusão:**

A transferência do nervo frênico foi eficaz na recuperação da flexão do cotovelo na maioria dos pacientes. Observamos sinais de neuroplasticidade que melhoraram o controle motor do braço ao longo do tempo. Os pacientes não apresentaram evidências de comprometimento pulmonar grave.

## Introduction

Brachial plexus injuries affect upper limb control and patient functionality. Total avulsion of the brachial plexus, occurring in 47 to 58% of trauma-induced cases, is particularly severe, resulting in complete loss of upper limb control. 1 Individuals suffering from such total traumatic brachial plexus injury (TTBPI) not only experience loss of functionality and autonomy but also frequently present posttraumatic stress disorder and suicidal ideation. 2 Moreover, TTBPI has economic repercussions at both individual and public expenditure levels. 3

In the absence of spontaneous recovery, several surgical treatments are available to attempt partial recovery of arm control, ranging from neurolysis to nerve transfers. Transferring an intact motor nerve to control other muscles is a viable and well-described approach capable of restoring elbow flexor control in those patients. 4 The Oberlin transfer, from the motor branch of the ulnar nerve to the motor branch of the biceps brachii, stands out as an effective method for elbow flexion recovery, 5 and it has become the preferred option in various services for the treatment of partial injuries.

In cases of total injury, however, the use of an extraplexual donor is necessary, since the ulnar nerve is damaged. The options include the phrenic, accessory spinal, and intercostal nerves. The phrenic nerve has been considered a viable option since the early reports by Russian surgeon Lurje 6 in 1948, and it is still a relevant option, 4 with encouraging reports of elbow flexion strength recovery. 7 8 9 However, there are concerns regarding the reports of associated diaphragmatic paralysis, evidenced by hemidiaphragm elevation, and compromised inspiratory strength, despite a lack of respiratory symptoms. 10 Forced vital capacity (FVC) may be an important index to evaluate these patients as it enables the comparison of respiratory function relative to what is expected given patient's age and sex. Values above 80% of the predicted are considered normal, while values up to 60% and 50% characterize mild and moderate disorders respectively, with values below 50% indicating severe disorders. 11 In addition to concerns about respiratory function, there are challenges regarding the motor control of the reinnervated biceps brachii due to the difference in the original function of the phrenic nerve, which may lead to involuntary contractions during respiratory effort in these patients.

Considering the severe implications of TTBPI, it is crucial to investigate surgical treatments that are both effective and safe. Phrenic nerve transfer appears to be a viable option to recover some level of elbow flexion, but the impacts on respiratory function and motor control remain uncertain. Thus, the aim of the current study was to evaluate elbow flexion strength production and respiratory function in TTBPI patients treated with phrenic nerve transfer to the motor branch of the biceps brachii.

## Materials and Methods

The present prospective case series included 16 patients who underwent surgery between 2014 and 2021 in the Microsurgery Service of our institution. The project was approved by an independent committee on human experimentation (under CAAE: 50087221.5.0000.5273). All patients signed the informed consent from before being enrolled in the study. The inclusion criteria were age over 18 years at the time of data collection, a postoperative period of more than 6 months, and absence of other orthopedic or neurological conditions affecting the upper limb.

**Fig. 1:**
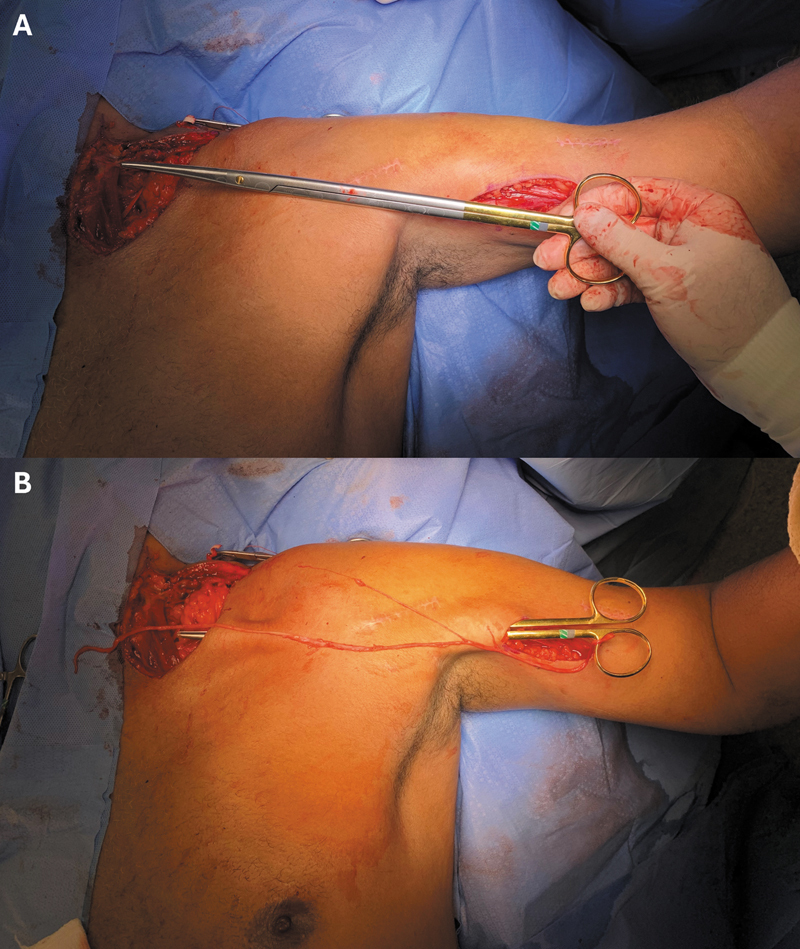
Planning ( **A** ) and construction ( **B** ) of the tunnel for the passage of the graft extracted from the sural nerve used in the transfer from the phrenic nerve to the motor branch of the musculocutaneous nerve.

The surgical procedure began with the patient in the supine position for graft harvesting from the sural nerve of the contralateral lower limb. Using a supraclavicular approach, the accessory nerve was transferred to the suprascapular nerve, and the phrenic nerve was dissected. The motor branch of the musculocutaneous nerve was accessed through a medial approach in the arm, and the graft was passed to the phrenic nerve through a tunnel prepared with a blunt scissor ( Fig. 1 ). Microsurgical sutures were then applied proximally and distally to the graft, followed by closure of the incision and placement of the upper limb in a sling. Sling immobilization was maintained for 3 weeks, after which the patients were referred to the Rehabilitation Department for joint stiffness control, improvement of postural stability, and promotion of limb motor control.

The patients were invited to participate in the project via telephone and email. All assessments were conducted during a single visit to the institute. The Disabilities of the Arm, Shoulder, and Hand (DASH) questionnaire was filled out by the patients, while elbow flexion strength was subjectively evaluated by a hand surgeon specialized in microsurgery using the Medical Research Council (MRC) scale. An objective assessment of elbow flexion strength was performed through maximal isometric contractions at 90° of elbow flexion using an isokinetic dynamometer (Humac Norm III, Computer Sports Medicine, Inc.).

**Fig. 2:**
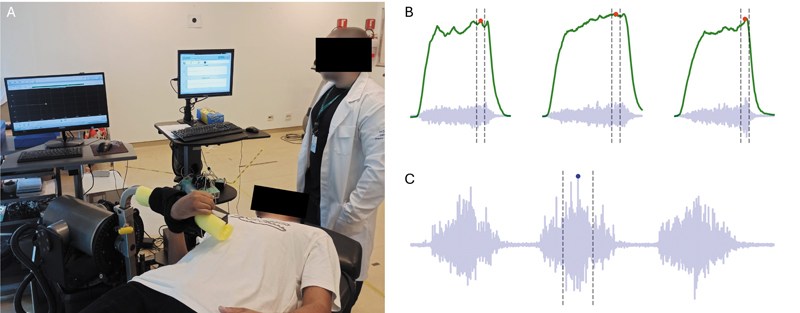
Assessment of elbow flexor muscle strength and activation of the biceps brachii ( **A** ). Representative signal of torque generated at the elbow joint (green line) and surface electromyography of the biceps brachii (blue line) during the three repetitions of the strength test ( **B** ) and forced respiratory cycles ( **C** ). The dashed vertical lines indicate the 500-ms period used to calculate the mean square root to quantify muscle activation.

For the dynamometer evaluation, the patients were positioned in the supine position with the arm beside the torso, and the hand was secured in the dynamometer handle according to the manufacturer's instructions ( Fig. 2A ). After familiarization with 3 submaximal contractions at 50% of maximum effort, 3 maximal contractions were performed with 30-second intervals. The maximum torque value across all contractions was recorded for analysis. This process was repeated with the patients performing maximal inspiratory and expiratory efforts in a randomized order before each maximal contraction. The healthy limb was tested first to facilitate understanding and minimize discomfort. Activation of the biceps brachii was monitored by electromyography (EMG) at 1 kHz (SAS1000; EMG system do Brasil) during maximal contraction. Biceps brachii activation was also monitored during three forced respiratory cycles. In both cases, the mean square root value in a 500-ms window around the EMG peak was used to quantify biceps brachii activation. The average activation across the three maximal contractions was used for analysis ( Fig. 2B ). Biceps brachii activation during forced respiratory cycles was expressed as a percentage of the activation observed during maximal contraction ( Fig. 2C ).

**Fig. 3:**
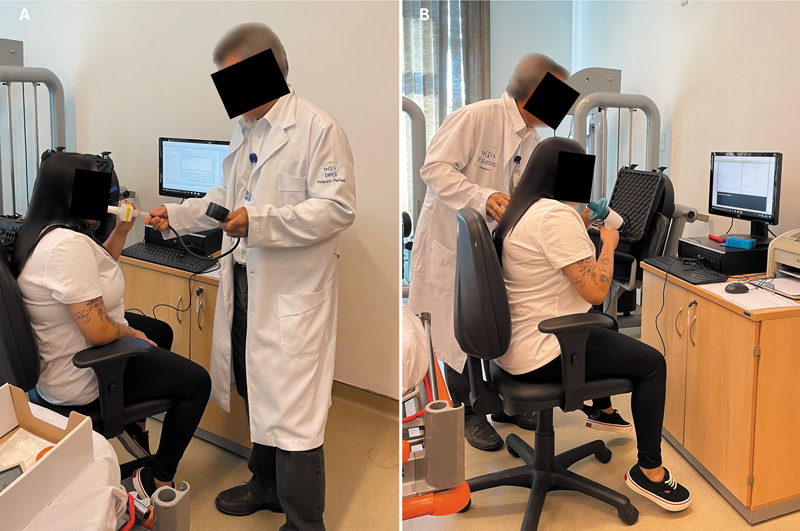
Assessment of respiratory function through the forced vital capacity (FVC), forced expiratory volume in the first second (FEV1), and Tiffeneau-Pinelli index (FEV1/FVC ratio; **A** ), along with the maximal inspiratory and expiratory pressures ( **B** ).

Respiratory function was assessed by FVC, forced expiratory volume in the first second (FEV1), and the Tiffeneau-Pinelli index (FEV1/FVC ratio), measured by an experienced pulmonologist using a Fleisch-type pneumotachometer (KoKo Sx1000, nSpireHealth) ( Fig. 3A ). Additionally, the maximal expiratory (MEP) and inspiratory (MIP) pressures were measured by the same professional using an analog manovacuometer (Wika) and expressed, as the FVC and FEV1, as a percentage of the values predicted for the Brazilian adult population 12 ( Fig. 3B ). Hemidiaphragm elevation, assessed by frontal plane chest radiography, was defined as an increase in the apex of the hemidiaphragm by two or more rib arcs above the anatomical position.

The numerical variables had a normal distribution according to the Shapiro-Wilk test, and they were expressed as mean ± standard deviation values. Differences in force production between respiratory maneuvers were tested by one-way repeated measures analysis of variance (ANOVA). Deficits in respiratory variables were verified by one-sample *t* -tests against the reference value (100%). The temporal trend of involuntary contraction throughout time was obtained by third-order polynomial regression with the respective coefficient of determination (R ^2^ ). All analyses were performed using customized Python 3.9 (free and open source) routines, and the α level was set at 0.05.

## Results

Among 41 eligible patients, 27 (65.8%) could be contacted, 16 (39%) of whom agreed to participate in the study, with demographic characteristics described in Table 1 . Respiratory function seemed to be partially affected compared with estimates in healthy individuals, although the respiratory pressures appeared to be preserved ( Table 1 ). Hemidiaphragm elevation was observed in 5 (37%) patients. All patients were injured in motor vehicle accidents, with all but one riding motorcycles. At the follow-up, no patient reported respiratory symptoms.

**Table 1:** Patient demographics

**Mean age (years)**	32 ± 10
**Male patients: n (%)**	13 (81.2%)
**Mean height (m)**	1.68 ± 0.10
**Mean weight (kg)**	68.0 ± 14.1
**Mean time between injury and surgery (months)**	7 ± 2
**Mean follow-up (months)**	39 ± 28
**Elbow flexion strength (MRC scale): n (%)**	
** 1**	1 (6.2%)
** 2**	2 (12.5%)
** 3**	6 (37.5%)
** 4**	7 (43.8%)
**Mean elbow flexion strength (%)**	9.1 ± 7.0
**Mean DASH score**	34 ± 11
**Mean FVC (%)**	81.1 ± 18.7*
**Mean FEV1 (%)**	79.1 ± 17.6*
**Mean Tiffeneau-Pinelli index**	1.0 ± 0.1
**Mean MEP (%)**	103.9 ± 36.9
**Mean MIP (%)**	108.1 ± 21.5

**Abbreviations:** DASH, Disabilities of the Arm, Shoulder, and Hand; FEV1, forced expiratory volume in the first second; FVC, forced vital capacity; MEP, maximal expiratory pressure; MIP, maximal inspiratory pressure; MRC, Medical Research Council.

**Note:** * Significantly lower than estimated in the healthy population, that is, 100% ( *p* < 0.05).

Elbow flexion strength ranged from 0.6% to 21.1%. The 5 cases (31%) of recovery above 10% happened more than 36 months after surgery. In total, 7 participants were classified as M4, 6, as M3, 2, as M2, and 1, as M1. We found no evidence that respiratory maneuvers before elbow flexion influenced force production (F2,24 = 1.04; *p* = 0.365).

**Fig. 4:**
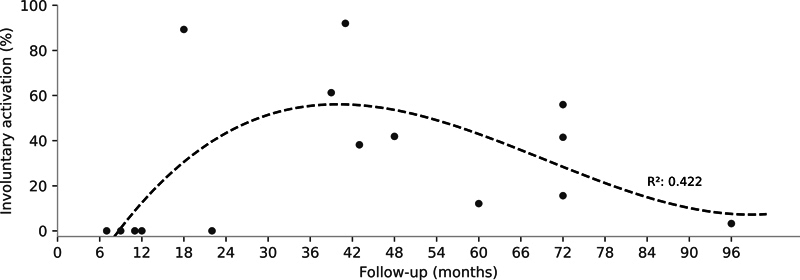
Involuntary contraction of the biceps brachii during forced respiratory cycles throughout the patients' follow-up period. The gray dots represent individual data points, and the dashed black line represents the trend derived from third-order polynomial regression, presented alongside the coefficient of determination (R ^2^ ).

Involuntary biceps brachii contractions during forced respiratory cycles were observed in 11 patients, predominantly in the first 12 months of follow-up. Subsequently, a level of activation of up to 91.9% was identified, followed by a decreasing trend over time ( Fig. 4 ).

**Fig. 5:**
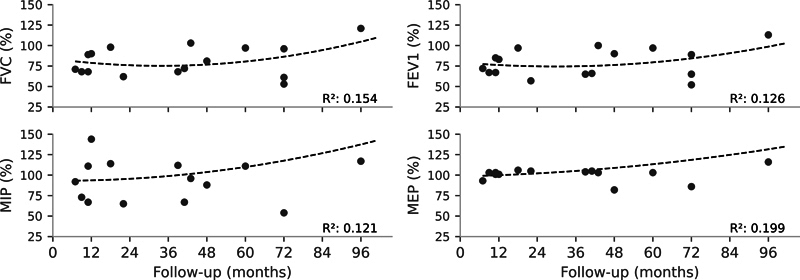
Longitudinal lung function throughout the patients' follow-up period. The gray dots represent individual data points, and the dashed black lines represent the trend derived from third-order polynomial regression, presented alongside the coefficient of determination (R ^2^ ). **Abbreviations:** FEV1, forced expiratory volume in the first second; FVC, forced vital capacity; MIP, maximal inspiratory pressure; MEP, maximal expiratory pressure.

Normal FVC values were observed in 8 patients (50%), while mild and moderate restrictive disorders were observed in 7 (43%) and 1 (6%) patient respectively. No patient presented severe restrictive disorder. Respiratory function parameters (FVC, FEV1, PImax, and PEmax) showed a relatively linear time trend ( Fig. 5 ).

## Discussion

Our main findings were that phrenic nerve transfer can recover elbow flexion at different levels, and that the extent of impairment in respiratory function is uncertain. Our sample consisted mainly of young male adults, victims of motorcycle accidents, which is consistent with the typical demographic profile reported for TTBPI patients globally. 1 7 10

Recovery of elbow flexion through phrenic nerve neurotization has been consistently demonstrated, corroborating our findings. In the present case series, we observed MRC strength levels up to grade III in 81.3% of the patients, similar to the recently reported 80% by Hussain et al. 13 and the 70% documented in the only systematic review found on the subject. 9 Recovery up to grade IV was observed in 43.8% of the patients, a rate comparable to the 62% reported by Socolovsky et al. 14 The time elapsed between injury and surgery in the current study averaged 7 months, slightly longer than the 4 to 5 months reported in other series, suggesting that phrenic nerve transfer may be applicable even with slightly-delayed treatments, as may be the case in public services and referral centers. However, caution is warranted, as it has been previously observed that surgeries performed within 4 months after injury resulted in grade-III MRC recovery in 96%, while later surgeries achieved only 43%. 9

While the MRC scale provides readily-available and useful information on muscle strength, quantitative measures are crucial to understand the magnitude of elbow flexion recovery. Sokolovsky et al. 8 14 provided the only objective reports of elbow flexion strength in TTBPI patients to date, with results ranging from 21 to 29% of the healthy limb, on average, after a minimum postoperative period of 10 months, with an average of approximately 36 months. Unfortunately, only averages were reported, 8 14 and we cannot assess the variability in strength recovery. However, based on our results and clinical practice, it is reasonable to expect a large variability, stemming from various factors, including patient conditions (such as extent of injury, time between injury and surgery, gender, or age) and surgical considerations (such as graft quality, procedural techniques, or team experience). Although the causes of variability are difficult to infer from available data, objective measures offer a quantitative assessment of elbow flexion recovery, contributing to documenting outcome evolution over time and across different technical approaches. Additionally, such measures provide a more comprehensive understanding of patient conditions, as achieving MRC grades up to IV 9 is commonly reported in this scenario, but expecting near-perfect recovery of control of elbow flexion is unrealistic for these patients.

We found inconsistent impairments in the patients' respiratory function, but no respiratory symptoms. This absence of symptoms is in line with findings from previous studies on phrenic nerve transfer. 8 13 15 16 Similarly, diaphragmatic paralysis has been previously observed without clinical manifestation. 10 The current study reported FVC and FEV1 values lower than expected in healthy individuals, reflecting a potential decrease in lung function, consistent with previous reports of 10% decrease in FEV from the preoperative period to 30 months postoperatively. 15 16 In the present study, we lack longitudinal data to track individual changes in respiratory function over time. However, it is reasonable to anticipate a recovery trend postsurgery if respiratory function was indeed affected. The absence of such a trend suggests that the observed variability in respiratory function may be influenced by other factors, such as decreased physical activity. Cadaveric studies 17 18 indicate that an accessory phrenic nerve may be present in 48 to 61% of individuals, which could explain why half of our patients exhibited normal FVC postsurgery. In brief, evidence of respiratory deficits is inconsistent and seems insufficient to cause respiratory symptoms.

An evaluation of muscle control is fundamental in neural transfer patients, as the original motor function may differ from the function of the reinnervated muscle. The phrenic nerve presents some advantages in the reinnervation of the the biceps brachii, such as the fact that it shares a common embryonic origin with the brachial plexus, it is easily accessible, and it is rich in myelinated fiber compared with other extraplexal options. 19 However, the intermittent nature of diaphragmatic activation differs from the expected elbow flexion patterns in everyday situations, 14 and involuntary activation of the biceps brachii may be observed in some patients during respiratory movements. 8 Our results show a temporal trend for this involuntary activation, which begins after an initial recovery period, probably due to graft healing, peaks in the early postoperative years, and then is controlled, reflecting the phenomenon of neuroplasticity. Interestingly, previous research 8 has observed that, in patients with elbow flexion recovery at level III of the MRC scale, maximum forced inspiration before elbow flexion improves force production compared with maximum forced expiration. In the present study, we did not observe differences related to respiratory maneuvers. Some patients in the current study presented lower levels of elbow flexion recovery. Thus, it is possible that such interference is accentuated in patients with better recovery, which deserves attention in future studies. Overall, these results highlight the central nervous system's ability to adapt to the new configuration of the phrenic nerve.

The present study has some noteworthy limitations. Isokinetic dynamometers may underestimate the strength of individuals unfamiliar with the equipment. We adapted the device to not require handle manipulation, facilitating handling of individuals with manual grip deficits, but equipment developed specifically for evaluation in this population may be more appropriate. Additionally, involuntary muscle activation was normalized by voluntary activation during maximum contraction. Normalizing it by the muscle's maximum intrinsic activation capacity, using M-waves obtained by electrostimulation, may be considered in future investigations.

## Conclusion

The phrenic nerve transfer effectively restored elbow flexion in most patients. While respiratory maneuvers did not influence force production, there was involuntary activation of the biceps brachii during forced respiratory cycles, peaking after an initial recovery period and subsequently declining. We found no evidence of severe pulmonary impairment in these patients, despite the signs of diaphragmatic paralysis.
